# Differences in Stress and Coping During the COVID-19 Pandemic in Families With and Without Children With Developmental Disorders or Chronic Conditions

**DOI:** 10.3389/fpubh.2021.704577

**Published:** 2021-08-20

**Authors:** Baiba Martinsone, Lilian Tzivian

**Affiliations:** ^1^Department of Psychology, University of Latvia, Riga, Latvia; ^2^Department of Medicine, University of Latvia, Riga, Latvia; ^3^Holon Institute of Technology, Holon, Israel

**Keywords:** COVID-19-related stress, coping strategies, children with developmental disorders, children with chronic conditions, parental stress

## Abstract

**Objectives:** To compare COVID-19-induced stress and coping in families with and without children diagnosed with developmental disorders or chronic conditions.

**Methods:** In this mixed-method design study, an online survey collected information on parental stress levels before and during COVID-19, sources of stress, and coping strategies using open-ended questions. Qualitative answers were categorized thematically. Multiple linear regression models were built for the association between changes in stress levels (during-before COVID-19) and sources of stress for parents of children of both groups.

**Results:** Answers of 1,827 parents were analyzed; of these, 186 (9.75%) had children with diagnosed problems. Changes in stress levels during vs. before COVID-19 were associated with the age of the parent, changes in working conditions, a total number of stressors, and distance learning of children. Stronger associations were found for parents of children with diagnoses. For example, for distance learning, the standardized beta (β) was 0.68 (95% confidence interval 0.37; 1.00) for parents of children without problems and β = 0.73 (0.43; 1.03) for those with problematic children.

**Conclusions:** Parents of children with developmental disorders need specific attention in a pandemic.

## Introduction

The Coronavirus Disease 2019 (COVID-19) global pandemic and restrictions resulting from it had a substantial impact on people and how they function in their daily lives, disrupting their usual routine, radically reducing social interactions, and increasing negative emotions and anxiety [e.g., (1, 2)]. Stressful events could cause discomfort or trigger a stress response, but they can also promote family strength and resilience (3). The COVID-19 related restrictions can be considered such a potentially stressful event.

Recent studies have addressed the stress associated with the COVID-19 pandemic [i.e., (4–9)]. Research has confirmed that during the COVID-19 crisis, parents experience stress regarding social distancing, remote learning, financial difficulties, and finding time and space for themselves (10, 11). The results of a recent study showed that the impact of COVID-19 quarantine on children's behavioral and emotional problems could be mediated by parental stress, both at an individual and couple levels, where the latter (couple stress) has the greater impact. Consequently, parents' stress and difficulties coping with quarantine restrictions can heighten problems in their children (10).

On the other hand, several recent studies have focused on coping strategies during the pandemic [e.g., (12–15)]. For instance, Taylor et al. (15) emphasized that reactions in response to the COVID-19 pandemic comprise multiple complex psychological factors, including coping and its intercorrelations with different forms of worry. Insight is needed into how individuals cope with stress—effectively or not effectively—to ensure that families are provided with the necessary support (16).

Caring for children with disabilities places the burden of caregiving on parents in normal daily circumstances [e.g., (17, 18)] and during the quarantine (19–22). During the pandemic, parents caring for children with special needs and disabilities could experience a low mood and distress (23). In particular, carers of children and adults with an intellectual disability reported significantly higher levels of mental health problems of their own in comparison with those carers whose children do not have an intellectual disability (24). Also, those parents who have pre-existing mental health issues of their own, dysfunctional families, and families that include people with post-traumatic stress disorders, anxiety, or depression, are particularly vulnerable in these situations (25). Researchers have established that there is essentially no difference between coping strategies in respondent groups with pre-existing anxiety and mood disorders and a normative sample (26). However, relatively few studies have focused on coping among those caring for children with disabilities.

This study aimed to compare COVID-19 first wave-induced stress and coping in families with and without children who have developmental disorders or chronic conditions. The study used mixed methodology providing the survey with open-ended questions for the main stressors and coping strategies. The main reason of such assessment was to provide participants with a possibility to express themselves and to uncover the stressors and coping strategies specific for this pandemic.

Specifically, the following research questions were posed:

1. What are the sources of stress caused by the COVID-19 pandemic emergency for parents of healthy children and for parents caring for children with developmental disorders or chronic conditions?
2. What coping strategies are used by parents of healthy children and parents caring for children with developmental disorders or chronic conditions?
3. Are there differences between the groups in sources of parental stress and coping strategies?
4. Which stressors and coping strategies explain differences between parental self-reported stress levels before and during the pandemic in both groups?

Based on the existing literature we hypothesized higher level of stress during pandemic in parents caring for children with developmental disorders or chronic conditions. We further hypothesized the existence of specific coping strategies that developed parents of children with developmental disorders or chronic conditions, which will differ from those that developed parents of healthy children. We hypothesized distance learning being the main stressor for both groups of parents, with its stronger association with changes in levels of stress due to the COVID-19 pandemic in the group of parents of children with developmental disorders or chronic conditions.

## Methods

### Study Design and Participants

The current study was a part of wider international comparable research “COVID-19 related stress and coping in families with children around the world,” which was led by the Seitoku University in Tokyo and involved researchers from Japan, Latvia, the United States of America, and Mexico. The study's main aim was to assess qualitatively the most important factors that increase COVID-19-induced stress in families with children in different countries and coping strategies used to overcome this stress. The Ethics Committee of Seitoku University in Tokyo granted permission for the research on 19/5/2020. The international study did not specifically focus on families with children with developmental disorders and chronic conditions. However, we considered it important to compare these two types of families in our Latvian sample. The reason for this decision was the insufficiency of studies making the accent on situation of families caring for such children in Baltic states and specifically in Latvia (27) and lack of appropriate recognition of problems of these families by countries' governments in previous years (28). For the Latvian part of the study, parents of children of any ages living in any area of Latvia that agreed to participate in the study were eligible for inclusion. Participants were invited to complete an electronic survey disseminated in two ways: by posting information with a link to the online survey in social networks (Facebook) and disseminating the link through municipal social services and local education authorities. Before they completed the survey, respondents gave informed consent to participate in the study, which was obligatory located at the first screen of the survey. Participants could not reach the survey without signing the informed consent electronically. Participants could quit the survey at any stage. Thus, participation in the study was voluntary. Data were collected during the first emergency due to the COVID-19 pandemics in May–June 2020.

### Study Questionnaire

The questionnaire was developed specifically for this research. It was comprised of open-ended questions (e.g., “What makes you feel stressed the most?” or “What activities or things are you doing to reduce your stress?”), and multiple-choice questions about the respondents' gender, age, family status, and occupation.

A separate question was devoted to one's stress level during the pandemic and the assessment of stress before the pandemic (both assessments were performed at the same time point). Participants self-assessed their level of stress in two-time points independently (before the pandemics and at the moment of the survey), using the Likert scale from 1 (not stressed at all) to 10 (very high level of stress). Having children with developmental disorders or chronic conditions was assessed using two specific questions: “does your child have a developmental disorders/chronic condition?,” with those answering yes also asked “Has this diagnosis been confirmed by a physician?.” The question about the kind of disorder was addressed in the survey but was not analyzed in this research.

### Thematic Analysis

Initially, thematic analysis was conducted on qualitative answers (on sources of stress and coping strategies) of the whole sample. The thematic analysis consisted of three stages:

1. Two researchers jointly categorized responses by themes. As each participant could answer the questions in a free format, they could answer differently, meaning the same problem, for example, answers to the question “What makes you feel stress the most” could be: “to take lessons with my children” or “to keep children near the computer during their studies.” These answers are related to the same theme: “necessity to help children with their studies.” Each participant could mention multiple stressors, and researchers manually evaluated all the responses identifying major themes.
2. When the number of themes no longer increased, the researchers independently coded the responses by grouping them into defined themes. Each theme became to be a dichotomous variable with “yes/no” categories. Any participant that mentioned the specific theme was classified by “yes” in the corresponding theme.
3. Coding results of both researchers were then compared, and any inconsistencies were discussed to arrive at a consensus. The inter-rater reliability of work performed by two researchers was 82%.

### Statistical Analysis

Descriptive statistics were performed for all study variables, including socio-demographic characteristics of study participants, their stress levels, and the main stressors they reported for the first wave of the COVID-19 pandemic. All stressors mentioned by each of the participants were summarized into continuous variable “number of stressors mentioned.” We further calculated the difference between self-reported stress levels during and before the COVID-19 pandemic by simple subtraction of provided scores, and that difference was our main outcome of interest.

We performed a univariate analysis for relationships between differences in stress levels and all study variables. We investigated differences between groups or parents of children with and without developmental disorders or chronic conditions. For comparison of the demographic parameters of participants we used Mann-Whitney test for continuous variables (e.g., age) and chi-square test for group variables (e.g., gender, place of residence, and occupation of the respondent).

We considered *p* < 0.05 as statistically significant at this stage of analysis.

Multiple linear regression models were built for the association between differences in stress levels and demographic parameters and stressors mentioned by parents. The full adjustment set was equal for both regression models (for parents of healthy children and for parents of children with developmental disorder or chronic condition) and included age of the parent, changed working conditions at the time of COVID-19, number of mentioned stressors, as well as individual stressors mentioned by the parents: work stress, financial stress, distance learning, necessity to help child with studies, everything needing to be done simultaneously, and fear of becoming ill. We built regression models independently for parents of children with and without developmental disorders and chronic conditions. Effect estimates (β) and 95% confidence intervals (CI) were presented in this analysis stage. All statistical analyses were performed using SPSS (26. Version) software (29).

## Results

### Study Participants

The research group comprised 1,827 participants; of these, 1,641 (89.8%) had healthy children, and 186 (10.2%) reported that their children had a developmental disorder or chronic condition. Most respondents were female (93.5%) and, during the pandemic, were primarily responsible for their children (79.4%). The mean age of participants was 39.30 (standard deviation 7.23 years). Most families had two children (43.5%), and 3.1% of parents had more than four children. Twenty per cent of parents were single. Most parents (69.5%) worked full-time. During the first wave of the COVID-19 pandemic, work conditions changed for 40.1% of the parents, but 33.1% continued their work the same as before the pandemic. The information provided by parents on the specific diagnosis of their children was used for cross-validation of the presence of diagnosis and was not analyzed independently. Most parents of children with developmental disorders or chronic conditions mentioned that their children have psychic, developmental and behavioral problems (39.2%), such as attention deficit and hyperactivity disorder, language development disorder, autistic spectrum disorders, schizophrenic conditions, intellectual disability. Another group of parents (17.7%) mentioned neurological disorders as the main problem of their children: movement disorders, shoulder nerve damage, or frequent migraines. Asthma and respiratory diseases were the third largest group of mentioned problems (8.1%). All other problems (e.g., endocrine, rheumatoid, gastric, and urological problems) were mentioned by between one and nine parents. Parents of healthy children and those with children with disabilities or chronic conditions differed in the number of children in the family (more parents with healthy children had one child only). No other significant differences in socio-demographic factors were observed between groups (Table 1).

**Table 1:** Descriptive statistics of study population of Latvian participants, by group.

**Variable**	**Categories**	**Parents of healthy children** **(*N* = 1,641)**	**Parents of disabled children** **(*N* = 186)**	***P-value^**#**^***
Age, median [25–75th percentile]		39.0 [34.0 – 44.0]	39.0 [35.0 – 45.0]	0.19
Gender, *N* (%)	Women	1,507 (92.9)	179 (96.8)	0.10
Place of residence, *N* (%)	Latvia	1,542 (99.2)	177 (98.9)	0.66
Occupation of respondent, *N* (%)	Maternity/paternity vacations Unemployed Housewife (both genders) Part-time work Full wage	92 (5.7) 81 (5.0) 101 (6.3) 200 (12.5) 1,130 (70.4)	12 (6.6) 11 (6.0) 12 (6.6) 29 (15.9) 118 (64.8)	0.59
Changed working conditions at the time of COVID-19, *N* (%)	Yes – work from home/ partly work from home/fired No Other	667 (40.6) 612 (37.3) 362 (22.1)	62 (33.3) 71 (38.2) 53 (28.5)	0.07
Being a single parent, *N* (%)	Yes	323 (19.7)	40 (21.5)	0.56
Number of children, *N* (%)	1 2 3 4 5 6	555 (34.0) 708 (43.4) 259 (15.9) 64 (3.9) 10 (0.6) 36 (2.2)	37 (19.9) 83 (44.6) 44 (23.7) 13 (7.0) 3 (1.6) 6 (3.2)	<0.01
Person responsible for children, *N* (%)	Respondent him/herself Partner Grandparents Other	1,299 (79.2) 104 (6.3) 102 (6.2) 136 (8.3)	148 (79.6) 10 (5.4) 14 (7.5) 14 (7.5)	0.84

# *p-value represents differences between parents of healthy children and parents of disabled children*.

### Thematic Analysis

In total, we were able to identify 16 categories of stressors and 14 categories of coping. In this paper, only those represented by more than 10% of participants were included in analysis. These were seven categories of stressors (physical and social distancing, work stress, financial stress, distance learning itself, necessity to help child with studies, everything needing to be done simultaneously, and fear of becoming infected) and four coping strategies (maintaining a routine, physical activities, spending time with the family, and personal time).

### Statistical Analysis

For both types of families (with and without children with disabilities) together, the level of stress during COVID-19 was higher than that before COVID-19: the median stress before COVID-19 was 3.00 (interquartile range, IQR 2.00–5.00), and the median stress during COVID-19 was 6.00 (IQR 4.00–8.00). There were significant differences in self-reported stress levels before (*p* = 0.04) and during (*p* < 0.01) the COVID-19 state of emergency between the parents of healthy children and parents of children with developmental disorders or chronic conditions, but not in the change of these two levels of stress.

We observed significant differences between the overall number of mentioned stressors in both groups, as well as in some of the stressors: social and physical distancing, distance learning, and necessity to help one's child with studies. These stressors were more often mentioned by parents of children with developmental disorders or chronic conditions. None of the coping strategies was significantly univariately related to the changes in stress levels. Groups did not differ with regard to reported stressors and coping strategies (Table 2).

**Table 2:** Stressors and coping strategies of parents with and without children with developmental disorders or chronic conditions during the COVID-19 pandemic first emergency situation in May-June 2020, by group.

	**Parents of healthy children** **(** ***N*** **=** **1,641)**		**Parents of disabled children** **(** ***N*** **=** **186)**		***P-*value*^**#**^***
	**No**	**Yes**	**No**	**Yes**	
**Stressors**, ***N*****(%)**					
Physical and social distance	1,124 (68.5)	107 (31.5)	517 (57.5)	79 (42.5)	<0.01
Work stress	1,184 (72.2)	457 (27.8)	123 (66.1)	63 (33.9)	0.09
Financial stress	1,383 (84.3)	258 (15.7)	147 (79.0)	39 (21.0)	0.07
Distance learning	1,161 (70.7)	480 (29.3)	114 (61.3)	72 (38.7)	<0.01
Necessity to help child with studies	1,410 (85.9)	231 (14.1)	147 (79.0)	39 (21.0)	0.02
Everything needing to be done simultaneously	1,387 (84.5)	254 (15.5)	151 (81.2)	35 (18.8)	0.24
Fear of becoming ill	1,345 (82.0)	296 (18.0)	151 (81.2)	35 (18.8)	0.76
**Coping strategies**, ***N*****(%)**					
Maintaining routine	1,366 (83.3)	273 (16.7)	151 (81.2)	35 (18.8)	0.47
Physical activities	545 (33.2)	1,096 (66.9)	60 (32.3)	126 (67.7)	0.87
Spending time with the family	1,300 (79.2)	341 (20.8)	143 (76.9)	43 (23.1)	0.45
Personal time	1,219 (74.3)	422 (25.7)	126 (67.7)	60 (32.3)	0.07
**Self-reported stress**					
Stress before Covid-19, median [25–75th percentile]	3.0 [2.0 – 5.0]		4.0 [2.0 – 6.0]		0.04
Stress during Covid-19, median [25–75th percentile]	6.0 [4.0 – 8.0]		7.0 [4.0 – 9.0]		<0.01
Difference of stress levels before and during COVID-19, median, mean ± SD	2.19 ± 2.65		2.22 ± 2.81		0.90
Number of stressors mentioned, median [25–75th percentile]	2.0 [1.0 – 3.0]		2.0 [1.0 – 4.0]		<0.01

# *p-value represents differences between parents of healthy children and parents of disabled children*.

Differences in self-reported stress levels for both groups of participants were significantly adversely associated with the age of parents and positively associated with changes in working conditions during the first wave of COVID-19, the number of stressors mentioned, and distance learning. Both models displayed statistical significance according to ANOVA: for parents of healthy children *F*_(9,1,621)_ = 14.3 (*p* < 0.01); for parents of children with developmental disorder and chronic condition *F*_(9,254)_ = 4.1 (*p* < 0.01). The percent of explained variance in differences in self-reported stress was higher for the group of parents caring of children with developmental disorder or chronic condition: adjusted *R*^2^ = 0.07 for parents of healthy children and adjusted *R*^2^ = 0.10 for parents of children with disabilities. Distance learning displayed the strongest association with changes in stress levels for both groups of parents, and this association was stronger for parents of children with developmental disorders or chronic conditions (Table 3).

**Table 3:** Differences in stress levels before and during COVID-19 pandemic emergency situation in May-June 2020, demographic parameters and stressors between parents with and without children with developmental disorders or chronic conditions—results of linear regression models.

**Variable**	**Parents of healthy children (** ***N*** **=** **1,641)**		**Parents of children with disabilities (** ***N*** **=** **186)**	
	**Standardized beta (β)**	**95% confidence interval**	**Standardized beta (β)**	**95% confidence interval**
Age^*^	−0.02	−0.40; −0.01	−0.03	−0.04; −0.01
Changed working conditions at the time of COVID-19*	0.37	0.20; 0.54	0.31	0.15; 0.47
Number of stressors mentioned*	0.17	0.04; 0.30	0.14	0.02; 0.26
Work stress	0.11	−0.22; 0.44	0.21	−0.11; 0.53
Financial stress	0.18	−0.19; 0.55	0.18	−0.17; 0.53
Distance learning*	0.68	0.37; 1.00	0.73	0.43; 1.03
Necessity to help child with studies	0.38	−0.10; 0.75	0.37	−0.03; 0.76
Everything needing to be done simultaneously	0.36	−0.87; 0.81	0.38	−0.04; 0.80
Fear of becoming ill	0.05	−0.30; 0.39	−0.04	−0.37; 0.29

* *Significant association presented in both groups of participants*.

## Discussion

In this study, we found that parents of children with developmental disorders or chronic conditions experienced more stress before and during the pandemic. Age of the parent, changes in working conditions of parents, number of mentioned stressors, and distance learning are associated with changes in stress levels before and during COVID-19 pandemic.

A situation of parents with children having developmental disorders or chronic conditions in Baltic States and specifically in Latvia is still under the influence of the late Soviet approach (27). Governments in these countries insufficiently recognize these problems as those need for specific attention, and do not provide additional help for families having children with developmental disorders or chronic conditions (28). For example, most of existing therapies are rather expensive, and state does not provide any compensation for it. This fact leads to a limited access of parents and children to these therapies (30). It is known that parents of children with developmental disorders or chronic conditions experience higher stress than those with healthy children (31, 32) and require higher levels of support from their families and professionals (33, 34). Lack of sufficient support and appropriate care might be a reason of higher stress of parents with children with developmental disorders or chronic conditions both before and during COVID-19 pandemic that was observed in this study.

In this study, we found as well that parents of children with developmental disorders or chronic conditions were more stressed from distance learning and the necessity to help a child with his/her studies than parents of children without problems. Most of studies performed in Baltic countries on school problems of children with developmental disorders or chronic conditions concentrated on the adaptation of these children in regular or special schools (33, 35, 36), and do not provide any information on parental feelings and experience during the educational process. Together with the insufficient governmental support in other issues related to caring for children with developmental disorders or chronic conditions, distance learning might become one of the main stressors for these parents.

The total number of children in the study with a condition diagnosed by a doctor was 9.75%, which corresponds with Raina et al. (17), who estimates that ~10% of all children have developmental disorders that require constant care and access to medical treatment that may be required until the child becomes an adult (17). Both parents of children in normative and clinical groups indicated various coping strategies that they use to reduce stress caused by the pandemic. The most frequently mentioned were: maintaining a daily routine (trying to stick with a standard routine and daily schedule), physical activities (engaging in sporting activities, working in the garden, walks in the fresh air), spending time with family (conversations, games or other activities), and time for oneself (for example, the parent devoting time to a hobby). The most frequently mentioned coping strategy for both groups were physical activities, followed by finding time for oneself to rejuvenate and spend time doing activities one enjoys.

Answering the second research question about differences in stressors and coping in both groups, a significant difference in frequency of responses was detected for two sources of stress. Respectively, parents of children with developmental disorders or chronic conditions more frequently reported that physical and social distancing were stressors, as was distance learning. Parents specifically reported they experienced difficulties because of the prohibition to meet with extended family members because playgrounds and sports fields were closed, after-school activities were suspended, and there were restrictions to meeting friends and other persons, for example, a psychologist. Drop-in or day care facilities were also not available either, which usually helped parents alleviate the burden of daily care needs. This is in line with the findings of other researchers which showed that parents of children with special education needs and disabilities reported a complete disruption of their daily routine, the loss of a support network, and the loss of the opportunity to receive treatment from specialists (23). Also, other research supports that carers of disabled children and adults face such difficulties as a feeling of abandonment and isolation as well as feeling on the verge of a breakdown (19–22).

A mother of three, whose oldest child is disabled and the nature of her work does not lend itself to working remotely, states:

“*The situation could somehow be solved because my husband was not working at the time and could look after all three children. I can't even imagine how it would have been possible to cope with the current situation if both parents had to work!”*

During the period of distance learning, children with special educational needs often did not receive the necessary support that was previously provided by the school. Therefore, parents often had to take on full responsibility for managing and supervising the teaching process. Although some research findings also show that remote learning can be beneficial to those children who experience difficulties at school (16, 23), still the need for parents to be involved in the education of a child with a developmental disorder or chronic condition caused significant stress, in comparison with parents with healthy children.

One mother's conclusion gets to the core of parents' feelings:

“*A primary school child with attention deficit disorder who is learning at home and work—two things that are, in principle, incompatible.”*

We did not find any differences between groups in coping strategies since both parents of healthy children and those who are raising children with developmental disorders or chronic conditions used similar methods to overcome challenges caused by stress during the pandemic. This conclusion corresponds with the findings of another recent study, wherein those adults with pre-existing anxiety-related disorders and mood disorders, even though their COVID-19 related stress was relatively higher, used similar coping strategies as the group without mental health issues (26).

To answer the third research question—which stressors and coping strategies explain differences between parental self-reported stress levels before and during the pandemic in both groups, we did not find the difference in changes in parental stress levels before and during the COVID-19 emergency situation in May–June 2020 in both groups. However, we observed significant differences in stress levels in these two groups both before and during the first wave of the pandemic, with higher stress levels for parents of children with developmental disorders or chronic conditions. The main stressor that affected the changes in stress levels was children's distance learning, and this stressor was stronger for parents of children of the clinical group than for parents of healthy children. We did not observe any relationship of coping strategies with changes in stress levels in the sample during and before the COVID-19 pandemic first wave in May–June 2020.

Factors that explain changes in the level of stress for both groups were the same—age of the parent, change in his/her working conditions, number of stressors that the parent mentioned, and distance learning of the child. Of these factors, the strongest association was found with distance learning, and this association was stronger in the group of parents of disabled children. We did not find any of the previously mentioned coping strategies to be significantly associated with changes in the level of stress. Regarding demographic variables, the changes in stress level can be explained by the age of the parent and the change in working conditions. Respectively, the older the parent, the less they felt stressed during the COVID-19 pandemic. Younger parents have greater stress in both groups, and that corresponds with findings in other studies [e.g., (14, 37)]. This could also be explained by the fact that, possibly, with age, adults develop greater emotional self-regulation (38). However, these claims about the relationship between pandemic stress and age are not unequivocal and possibly could be associated with socio-cultural aspects or situation related to health policy (39). The stress of the participant was higher in those that did not have changes in their working conditions during COVID-19 in comparison to those that started to work from home. In addition, this association was slightly stronger in the group of parents who are raising healthy children.

Changes to the level of stress can be explained by the number of stressors; that is, the more stressors experienced by parents during the pandemic state of emergency, the greater the change in self-reported stress levels before the pandemic and during the state of emergency. This association is also slightly more evident in the group of parents of healthy children, even though parents with healthy children were more likely to only have one child, in comparison with the clinical group of parents, who were likely to be raising two or three children. This could possibly be illustrated by one mother's answer about the multi-tasking experienced and simultaneous sources of stress that may also influence a family with healthy children:

“*Stress is caused by the fact that you are home all the time and you cannot be alone. That you constantly have to prepare meals and in addition to working remotely, you have to attend to household tasks. That family members don't respect that I, too, have to work and expect that because I am home, that I am accessible. A sense of guilt that I'm not doing either job properly. Uncertainty about the length of time these restrictions will be in force.”*

Of the stressors mentioned, changes in parental stress during the pandemic were explained by distance learning, and greater influence on the increase in stress was apparent in the group of parents with children with developmental disorders or chronic conditions. Although the standardized betas of the association between distance learning and changes in levels of stress during COVID-19 pandemic was stronger in parents of children with developmental disorders or chronic conditions in comparison with parents of healthy children, these differences were non-significant. Larger studies are needed to proof the observed associations. Nevertheless, based on the results of this study, public health authorities and policymakers can provide some practical changes that will have an impact on the quality of life of parents with children with developmental disorders and chronic conditions. Children in such families should be provided with special support during their distance learning, giving the parents time for themselves to cope with stress. In addition, policymakers should consider the minimization of stress induced by physical and social distance and change of working conditions that were the main factors that were mentioned by parents of children with developmental disorders or chronic conditions. It is especially important for parents of older ages that can be provided by consultations of professionals and support groups organized by the state authorities.

It is important to emphasize that in both groups of parents, the self-reported increase in stress does not differ, whereas, in the group of healthy children, the baseline and pandemic-related stress is lower than in the clinical group. This leads us to the conclusion that the contribution of the pandemic to changes in stress levels in families with children is equal, however for parents of children with developmental disorders or chronic conditions, the overall stress is significantly higher both before and during the COVID-19 pandemic state of emergency. Most of studies on children with developmental disorders or chronic conditions until now were performed in Lithuania (27), leaving specific Latvian conditions out of the scope. More studies are needed to start an evidence-based dialog with state authorities about the appropriate help and treatment for parents of such children. Future programs and subsequent interventional research suggesting and implementing different coping strategies should be planned at the governmental level to provide parents with specific techniques that will help them to overcome pandemic-induced problems.

## Limitations and Strengths

Limitations can be attributed to the online survey that, possibly, may not have reached those groups of respondents who do not have access to technologies. Another limitation could be the comparatively low participation of single parents (research shows that there are more than 20% of single-parent families in Latvia). Although the percentage of children with a diagnosed condition corresponded with the general distribution of disorders, the sample in this study had a comparatively low number of parents with children with diagnosed problems, and this could limit the generalization of findings. Also, our study had a lack of possibility to address the results of the study to the specific diagnosis of the child as the information provided by parents on it was insufficient. In this manuscript, we also did not provide a proper analysis of the qualitative part of the study. We used qualitative answers of participants as a basis for the quantitative part. In addition, we assessed the stress of participants using one Likert-scale question, and memory bias might affect the answers of participants concerning their level of stress before COVID-19 pandemic.

The strength of this study is that it was conducted during the state of emergency brought about by the pandemic, thereby mostly avoiding retrospective reports. Second, this study used a quantitative-qualitative survey, not rating responses on how they conform within the parameters of a construct but openly asking about sources of parental stress and coping strategies. The mixed-method design of the study provided the opportunity to “hear the voice of the parents” and gain rich data on the experiences of families with children during the COVID-19 pandemic.

## Data Availability Statement

The qualitative data is not available as permission to provide secondary data analysis was not sought in the information to participants prior to consent. The quantitative data could be provided upon request.

## Ethics Statement

The studies involving human participants were reviewed and approved by the Ethics Committee of Seitoku University in Tokyo granted permission for the research on 19/5/2020. The patients/participants provided their written informed consent to participate in this study.

## Author Contributions

Both authors listed have made a substantial, direct and intellectual contribution to the work, and approved it for publication.

## Conflict of Interest

The authors declare that the research was conducted in the absence of any commercial or financial relationships that could be construed as a potential conflict of interest.

## Publisher's Note

All claims expressed in this article are solely those of the authors and do not necessarily represent those of their affiliated organizations, or those of the publisher, the editors and the reviewers. Any product that may be evaluated in this article, or claim that may be made by its manufacturer, is not guaranteed or endorsed by the publisher.
